# Determinants of plant community along environmental gradients in Geramo forest, the western escarpment of the rift valley of Ethiopia

**DOI:** 10.1371/journal.pone.0294324

**Published:** 2023-11-27

**Authors:** Zeleke Assefa Getaneh, Sebsebe Demissew, Zerihun Woldu, Ermias Aynekulu

**Affiliations:** 1 Department of Plant Biology and Biodiversity Management, College of Natural and Computational Sciences, Addis Ababa University, Addis Ababa, Ethiopia; 2 Department of Biology, College of Natural Sciences, Arba Minch University, Arba Minch, Ethiopia; 3 World Agroforestry (ICRAF), United Nations Avenue, Nairobi, Kenya; Technical University in Zvolen, SLOVAKIA

## Abstract

Detailed information on plant community types, distribution, and their relationships with various environmental gradients is crucial for understanding forest dynamics and sustainable forest management because plant community types are influenced by various environmental factors. Thus, this study was conducted to investigate plant community types and species diversity in relation to various environmental gradients in Geramo Forest, which is a remnant forest in the western escarpment of the Rift Valley of Ethiopia. Vegetation data were collected in 96 nested plots (20 × 20 m^2^ and five 1 ×1 m^2^) laid systematically at a distance of 250 m along 16 line transects, which were laid 300 m apart. Environmental and disturbance variables were also collected from each main plot. Agglomerative hierarchical cluster analysis and Canonical correspondence analysis (CCA) with R software were used to identify plant community types and analyze the relationship between plant community types and environmental variables, respectively. The Shannon Wiener diversity index was used to compute species diversity among community types. Five significantly different (p ≤ 0.001) plant community types were identified. The CCA results showed that species diversity and community composition among different community types were significantly influenced by altitude, disturbance, soil organic carbon, slope, soil available phosphorus, and pH, which revealed the compounded effect of various environmental factors on species richness, diversity, and evenness among plant community types. The study also identified a significant level of anthropogenic disturbance and a strong reliance of the local community on the forest in the research area. Therefore, it is recommended that sustainable forest conservation interventions be implemented through awareness creation and the promotion of community-based approaches.

## Introduction

Long-term and effective management of forest resources requires the classification of natural ecosystems into potential plant communities and habitat types and an understanding of species-environmental relationships along environmental gradients [1, 2]. Furthermore, understanding forest dynamics and sustainable management of forest resources entails detailed information on plant species composition, diversity, distribution, plant community types, and their relationship with various environmental factors [2] so as to develop and implement sustainable forest management activities to conserve the forest and its associated biodiversity from escalating anthropogenic pressure. Plants assemble in communities, and these plant communities are strongly influenced by various temporal and spatial patterns and environmental variables that can affect the distribution and structure of plants within the community [3, 4]. Interactions between plants and their environment create a continuously changing mosaic of vegetation types across the landscape. Environmental gradients are the driving forces behind the distribution of plant species, as they influence the species’ physiological and ecological needs. Thus, understanding the relationships between plant communities and environmental gradients and how these communities function and respond to changes in environmental factors is crucial for predicting the effects of climate change on vegetation patterns and ensuring their long-term sustainability.

Biotic and abiotic factors (topography, land use changes, soil factors, climate changes, competition, herbivory, etc.) have a great influence on plant composition, diversity, and their spatial distribution patterns [5, 6]. Biotic factors, such as competition for resources and interactions with other organisms, play a crucial role in determining the distribution of plant communities and can directly influence the presence or absence of certain species within a given area. For instance, competition for sunlight, water, and nutrients can limit the growth and survival of some plant species while favoring others that are better adapted to the prevailing conditions. Interactions with other organisms, such as herbivory or mutualistic relationships, can also shape plant community distributions by influencing the abundance and distribution of particular species [7]. Altitude is one of the decisive topographic (abiotic) variables in determining the patterns of plant species distribution and plant community formation by causing variations in atmospheric pressure, humidity, temperature, soil type, and microclimatic conditions, which determine the patterns of species by determining the growth and development of plants [1, 8, 9]. Slope again has a strong contribution in determining plant distribution by causing the accumulation and export of soil nutrients [10]. Edaphic and disturbance factors also affect the pattern of plant species distribution and community formation [8, 11].

The existence of a wide range of altitude and various geographical features in the Ethiopian landscape has helped the emergence of a wide range of habitats that are suitable for the evolution and survival of various plant and animal species [12, 13]. These variations in topographic and geographical features in Ethiopian landscapes resulted in the existence of various vegetation types and high species diversity (over 6000 species of higher plants with 10% endemic species) [14], which indicates the correlation between plant species composition, diversity, and environmental gradients such as altitude, slope, aspect, and soil physicochemical features [2, 15]. Several findings also depicted the significant impact of topographic and soil variables on plant species distribution and community formation [16–19].

However, various anthropogenic activities such as agricultural intensification, deforestation, forest degradation, overutilization, and settlements have caused severe threats to the forest resources of Ethiopia [13]. As a result, only small natural forest patches remained in the low and highlands of the southern and southwestern parts of the country, which still have not escaped from the mounting anthropogenic pressure. The Geramo forest, as one of the natural remnant forests of the country, is also subjected to the aforementioned anthropogenic pressures and is under serious anthropogenic threat. Thus, understanding plant species diversity, community types and their species distribution patterns along the environmental gradients would provide relevant information for effective and sustainable management of this forest. In view of this, the present study aims to (i) identify plant community types and species diversity and (ii) assess the relationships between plant community types and environmental factors in the Geramo forest, the western escarpment of the Rift Valley of Ethiopia.

## Materials and methods

### Description of the study area

**Fig 1:**
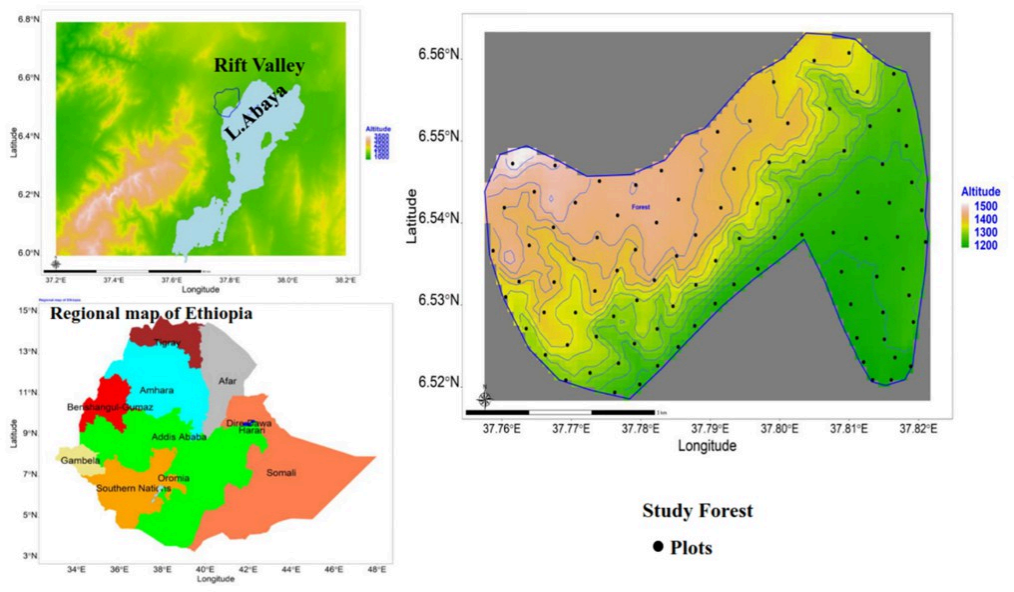
Location map of the study area. This work does not need to supply a copy right notice for Fig 1 because the shape file of Fig 1 (location map of the study area) is free and open to researchers and downloaded from the Ethiopian Mapping Agency website (https://africaopendata.org/dataset/ethiopia-shapefiles).

Geramo forest is located between 6° 30’ 29.88"–6° 34’ 59.88" N and 37° 43’ 59.88"–37° 49’ 0.12" E along the western escarpment of the southern part of the Rift Valley in Southern Ethiopia (Fig 1). It lies in an elevation range between 1188 and 1501 m.a.s.l. and covers an area of approximately 2104 hectares. The topography of the study area is a nearly flat plain around the coastal strip of Lake Abaya (one of the Rift Valley lakes in Ethiopia) and mountainous, highly undulating, and rugged around the top of Geramo forest. There was no specific permission required to conduct this study in the area since the study did not involve illegal actions like the extraction of endangered species. However, to access the study forest and collect related field data, permission was obtained from the Agriculture and Rural Development office of the Mirab Abaya district with a support letter from Addis Ababa University, College of Natural and Computational Sciences, Department of Plant Biology and Biodiversity Management. According to the most recent and improved classification of the potential vegetation of Ethiopia and Eritrea, the vegetation of the Rift Valley part of the study area belongs to the *Acacia*–*Commiphora* woodland and bushland proper (ACB) [20].

#### Climate

Three main agro-ecological zones, Dega (highland), Woina-Dega (mid-altitude) and Kolla (lowland), were recognized in Mirab Abaya woreda, each accounting for 11%, 27%, and 62% of the total land area in the respective order [21]. Among these, the study area is found in the low altitudinal range, characterized by moderately hot and dry climatic conditions with low precipitation.

Based on the climate data of the nearest town (Mirab Abaya) obtained from the National Meteorological Services Agency from 1991 to 2019, the study area receives a bimodal rainfall distribution with an average annual precipitation of 790 mm. The two wet seasons are interrupted by two dry seasons in the area; the two wet seasons lie between March/April–May and September–November. The minimum and maximum monthly temperatures of the area were 16.4°C and 33.4°C, respectively, with an average annual temperature of 24.4°C.

### Data collection

#### Sampling design

A systematic sampling technique was employed for vegetation and environmental data collection to ensure full coverage of environmental variation and habitat heterogeneity [22]. A total of 96 sample plots along 16 line transects were laid using GPS. The transects were 300 m apart and the sampling plots were 250 m apart from each other. A square plot of 20 x 20 m (400 m^2^) was used to collect data on woody species. Five 1 x 1 m (1 m^2^) subplots (four at each corner and one in the center) were nested in each 400 m^2^ main plot to collect data on herbaceous species.

#### Vegetation and environmental data collection

The percent canopy cover of the trees, shrubs, and lianas was visually estimated. We recorded the name (local/ scientific) of the species and measured the height and diameter of trees and shrubs with diameter at breast height (DBH) ≥ 2 cm and height ≥ 1.5 m using a caliper and clinometer/calibrated stick, respectively. The percent cover of the herbaceous species was also estimated in the small plots nested in the larger ones. Environmental variables, including altitude, slope, and geographical coordinates of each plot, were measured using GPS. Anthropogenic disturbances (cutting trees/for firewood, fencing, house building/, charcoal production, grazing, forest fire and grass cutting) were noted in each plot. The type and level of disturbance were determined using a 0–3 subjective ordinal scale as: 0 = nil; 1 = low; 2 = moderate; and 3 = heavy, following [8]. Plant specimens were collected, numbered, dried and placed in a reference collection following standard herbarium procedures. The specimens were then identified by comparing them with specimens already identified in the National Herbarium of Ethiopia (ETH) and by referring to the flora of Ethiopia and Eritrea published in volumes 1–8.

Soil samples were collected from each of the five 1 m^2^ subplots (4 at the corner & 1 at the center of each main plot) using a soil auger at a depth of 30 cm [23]. The soil samples were mixed and a composite sample (about 100g) of each main plot was taken to the laboratory for analysis. In the laboratory, the composite soil samples were air dried, grinded and sieved using a 2 mm sieve. Then the physicochemical parameters of each soil sample were determined in the soil laboratory of Arba Minch University following standard procedures [24]. Accordingly, soil texture was determined using the Bouyoucos Hydrometer method, organic carbon was measured using the [25] method, pH was measured using a pH meter, electrical conductivity was measured using a conductivity meter, total nitrogen was measured using the Kjeldhal procedure, cation exchange capacity was determined using the ammonium acetate and available phosphorus was determined using the Bray II method.

### Data analysis

#### Plant community classification and species diversity

We used agglomerative hierarchical clustering analysis with similarity ratio as a resemblance index and the “Ward method" as a classification technique using R software version 4.2.2 [26] to classify plant community types. The optimal number of clusters and, the correlation between species distribution and environmental variables were determined using R software version 4.2.2 [26]. To determine the characteristic species used to name the community types of the study forest, a synoptic table analysis was performed. Then, based on the synoptic cover abundance values, three characteristic species with high synoptic cover abundance values (mean frequency x mean cover-abundance) were used to name the plant community types following the method of van der Maarel et al. [27]. We used the Multiple Response Permutation Procedure (MRPP) using R software version 4.2.2 [26] to test whether there is significant difference between the clusters identified using the agglomerative hierarchical classification of sampling units.

Detrended correspondence analysis (DCA) of vegetation data was carried out prior to determine the appropriate method used in this analysis) using R software version 4.2.2 [26], and the DCA result revealed that the unimodal method should be used for analysis. So, Canonical Correspondence Analysis (CCA) was applied as a direct ordination method to determine the relationship between plant community types and environmental variables) using R software version 4.2.2 [26].

A Kruskal–Wallis test followed by Dunn’s post-hoc test was performed to check whether the variation within plant community types was significant or not with regard to non-collinear significant environmental variables) using SPSS 20.

Species richness, Shannon-Wiener diversity and evenness indices were computed using free statistical R software version 4.2.2 [26]. We used Shannon-Wiener’s diversity index to measure species diversity, because it considers both species richness and evenness into account and is not affected by sample size [22, 28]. It was calculated using the following equation.

$$Shannon\text{-}Weiner\;diversity\;index\;\left(H’\right)=-\sum_{i=1}^{S}pi\;\text{ln}\;pi$$

Where, H^’^ = Shannon-Wiener diversity index, Pi = the proportion of individual or the abundance of i^th^ species expressed as a proportion of total cover in the sample, lnPi = log_e_Pi, S = the number of species.

$$Equitability\;or\;evenness\;\left(J\right)=\frac{H\prime }{\text{ln}\left(S\right)}\;or\;=\frac{H\prime }{H\;\text{max}}$$

Where, H^’^ = Shannon-Wiener Diversity Index, H’_max_ = lnS = the natural logarithm of the total number of species, S = total number of species in the sample.

## Results

### Plant community types

In total, 180 plant species belonging to 54 families and 132 genera were identified in the Geramo forest and surrounding areas. The majority of plant species were herbs (35%), followed by trees (27%), shrubs (26%) and climbers (12%). The list of all identified plant species is given in the S1 Table. The family with the highest number of species was Poaceae, which consisted of 29 species (16% of the total). This was closely followed by Fabaceae with 19 species (11% of the total) and Euphorbiaceae with 9 species (5% of the total). Furthermore, 23 families were represented by a single species.

**Fig 2:**
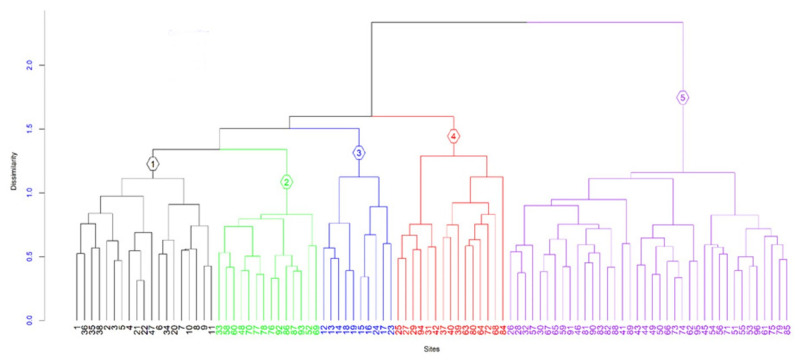
Dendrogram showing the five plant community types. 1 (Community type 1) = *Dichrostachys cinerea-Balanites aegyptiaca-Commiphora africana* community type, 2 (Community type 2) = *Terminalia brownii*—*Hyparrhenia filipendula*—*Rhus natalensis* community type, 3 (Community type 3) = *Vachellia nilotica*—*Harrisonia abyssinica*—*Ziziphus mucronata* community type, 4 (Community type 4) = *Senegalia brevispica*—*Croton zambesicus*—*Teclea nobilis* community type, 5 (Community type 5) = *Combretum collinum*—*Heteropogon contortus*—*Grewia velutina* Community type.

Five plant community types were identified in the study area (Fig 2). There was a statistically significant difference (*R = 0*.*569*, *p ≤ 0*.*001*) in floristic compositions among the five plant community types based on the Multi-response Permutation Procedures (MRPP) test. The plant communities were named after the three characteristic tree or shrub species based on their average synoptic cover abundance values in each group (Table 1, S2 Table). Each identified community type is described below.

**Table 1:** Synoptic cover abundance value of species in each community type.

Species	C 1	C 2	C3	C 4	C5
***Dichrostachys cinerea***	**5.05**	5.01	3.4	3	4.26
***Balanites aegyptiaca***	**3.79**	2.57	3.62	1.67	3.21
***Commiphora africana***	**3.53**	3.41	3.49	2.27	1.05
***Terminalia brownii***	3.26	**7.64**	1.5	3.37	4.26
***Hyparrhenia filipendula***	2.53	**7.14**	4.4	2.73	4.30
***Rhus natalensis***	3.01	**6.36**	4.3	3.93	4.29
***Vachellia nilotica***	3.11	5.64	**6.0**	1.87	1.76
***Harrisonia abyssinica***	2.05	4.86	**4.9**	1.27	4.19
***Ziziphus mucronata***	2.68	1.57	**4.5**	1.13	2.21
***Senegalia brevispica***	0.53	0.57	1	**4.8**	0.95
***Croton zambesicus***	0.53	0	0.5	**3.8**	0.18
***Teclea nobilis***	1.79	0.86	0	**3.47**	2.47
***Combretum collinum***	0.21	0.64	0	1.53	**7.37**
***Heteropogon contortus***	2.42	5.05	1.5	3	**5.18**
***Grewia velutina***	1.26	3.79	0.7	1.47	**4.32**
**Number of Plots**	19	14	10	15	38

C1 (Community type 1) = *Dichrostachys cinerea-Balanites aegyptiaca-Commiphora africana* community type, C2 (Community type 2) = *Terminalia brownii*—*Hyparrhenia filipendula*—*Rhus natalensis* community type, C3 (Community type 3) = *Vachellia nilotica*—*Harrisonia abyssinica*—*Ziziphus mucronata* community type, C4 (Community type 4) = *Senegalia brevispica*—*Croton zambesicus*—*Teclea nobilis* community type, C5 (Community type 5) = *Combretum collinum*—*Heteropogon contortus*—*Grewia velutina* Community type. Values in bold indicate the synoptic value of characteristic species used to name each plant community type.

A. Dichrostachys cinerea-Balanites aegyptiaca-Commiphora africana community type (C1).

This community type is found in an altitudinal range between 1214–1262 m.a.s.l. The dominant and characteristic tree and shrub species of this community include *Dichrostachys cinerea*, *Balanites aegyptiaca*, *Commiphora africana*, *Grewia bicolor*, *Euclea divinorum*, *Tamarindus indica* and *Canthium pseudosetiflorum*. *Barleria eranthemoides* was the dominant herbaceous in this community type. The lianas species such as *Cissus quadrangularis*, *Kleinia squarrosa* and *Sarcostemma viminale* were also abundant in this community type.

*B*. *Terminalia brownii*—*Hyparrhenia filipendula*—*Rhus natalensis community type (C2)*. This community type is found in an altitudinal range between 1222–1350 m.a.s.l. The dominant and characteristic tree and shrub species of this community include *Terminalia brownii*, *Rhus natalensis*, *Sclerocarya birrea*, *Ximenia americana*, *Commiphora schimperi*, *Commiphora habessinica*, *Stylosanthes fruticosa* and *Vachellia seyal*, *whereas the dominant liana was Ampelocissus bombycina*. The most dominant species in the herbaceous layer include *Hyparrhenia filipendula*.

*C*. *Vachellia nilotica*—*Harrisonia abyssinica*—*Ziziphus mucronata community type (C3)*. This community type is found in an elevational range between 1199–1223 m.a.s.l. The dominant and characteristic tree and shrub species of this community type include *Vachellia nilotica*, *Harrisonia abyssinica*, *Ziziphus mucronata*, *Senegalia senegal*, *Vachellia tortilis*, *Balanites rotudifolia* and *Allophylus rubifolius while the dominant liana was Cissus rotundifolia*. The most dominant species in the herbaceous layer include *Brachiaria deflexa*, *Commelina diffusa* and *Cynodon dactylon*.

*D*. *Senegalia brevispica*—*Croton zambesicus*—*Teclea nobilis community type (C4)*. This community type is found in an altitudinal range between 1235–1407 m.a.s.l. The dominant and characteristic tree and shrub species of this community include *Senegalia brevispica*, *Croton zambesicus*, *Teclea nobilis*, *Pappea capensis*, *Combretum mole*, *Acalypha fruticose* and *Boscia angustifolia* while the dominant lianas include *Rhynchosia stipulosa* and *Ampelocissus schimperiana*. The most dominant species in the herbaceous layer include *Phragmites karka*, *Panicum monticola* and *Actinopteris semiflabellata*.

*E*. *Combretum collinum*—*Heteropogon contortus*—*Grewia velutina community type (C5)*. This community type is found in an altitudinal range between 1245–1439 m.a.s.l. The dominant and characteristic tree and shrub species of this community include *Combretum collinum*, *Heteropogon contortus*, *Grewia velutina*, *Dodonaea angustifolia*, *Lannea schimperi*, *Mystroxylon aethiopicum*, *Osyris quadripartita*, *Flacourtia indica* and *Ozoroa insignis* while the dominant liana was *Jasminum grandiflorum*. The dominant species in the herbaceous layer include *Sporobolus ioclados* and *Melinis tenuissima*.

### Plant communities along the environmental gradient

#### Canonical correspondence analysis (CCA)

**Fig 3:**
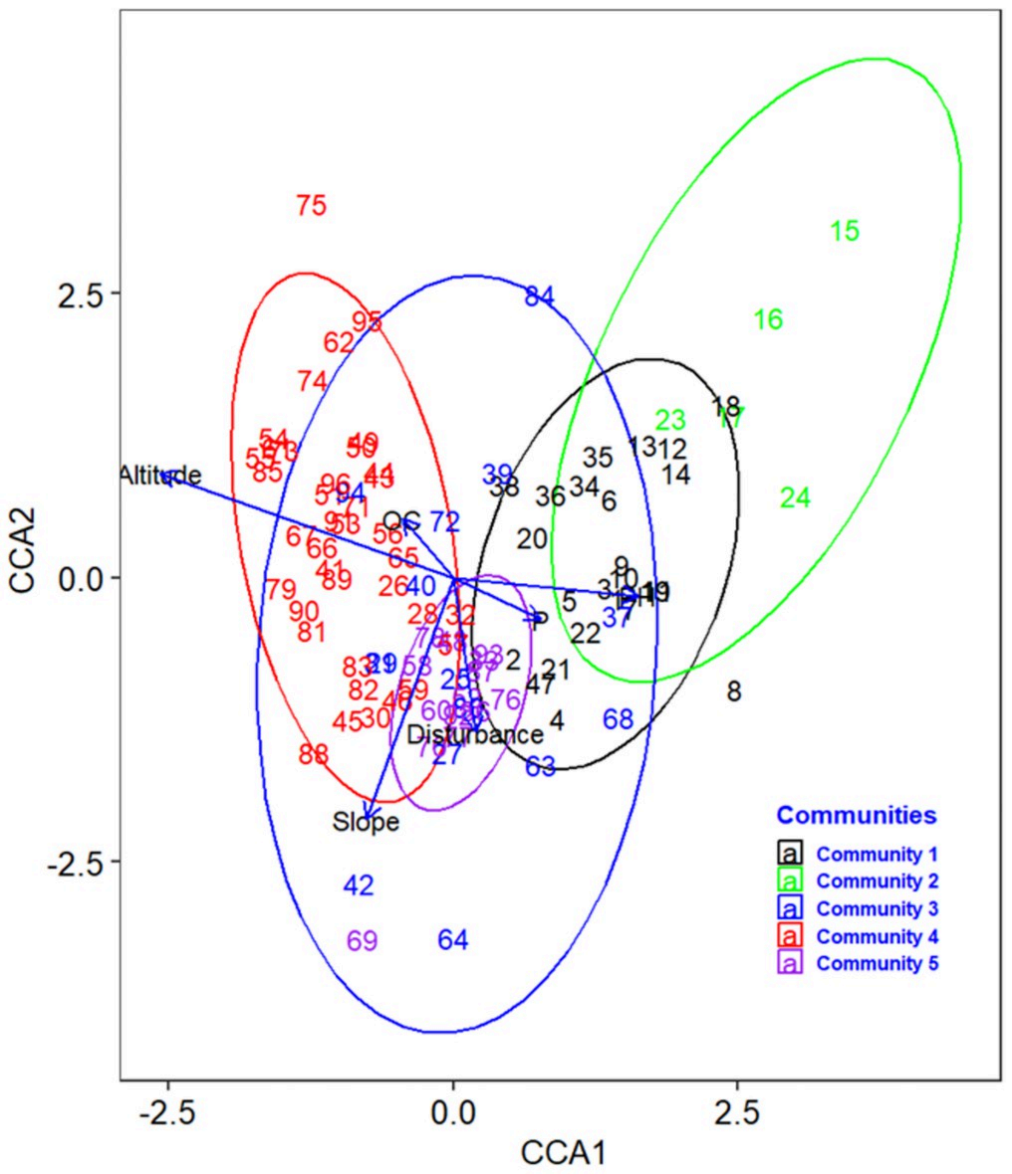
CCA biplot of community types with significant environmental variables (*p*≤0.05).

In this study, a canonical correspondence analysis (CCA) was utilized to examine the relationships between community types and environmental variables, following the results of the DCA analysis. Through the application of an automatic forward and backward selection process using the Monte Carlo test (with Adonis function), it was determined that out of the thirteen environmental variables initially considered, only six variables (altitude, slope, disturbance, soil organic carbon, available phosphorus, and pH) had a significant impact on the composition and distribution of plant communities (p ≤ 0.05) (Table 2 and Fig 3). Specifically, community type 1 exhibited a strong association with pH and available phosphorus concentration in the soil samples in the corresponding plots, while community type 2 displayed a minor association with soil pH. Disturbance, organic carbon content (OC) of the soil samples, and slope were found to be influential factors for community type 3. Altitude emerged as a major influencing factor for community type 4, while disturbance played a key role in community type 5. The findings from the CCA analysis reinforce the outcomes of the cluster analysis, indicating that both methods complement each other.

**Table 2:** Result of Monte Carlo test using function Adonis of environmental variables.

Variable	Df	Sums of Sqs	Mean Sqs	F. Model	R^2^	P value
**Altitude**	1	2.0691	2.06912	12.3885	0.10823	0.001 ***
**Slope**	1	0.5641	0.56405	3.3772	0.0295	0.001 ***
**Aspect**	1	1.1854	0.19757	1.1829	0.06201	0.113
**Disturbance**	1	0.2845	0.2845	1.7034	0.01488	0.042 *
**OC**	1	0.3772	0.37725	2.2587	0.01973	0.005 **
**avP**	1	0.3549	0.35491	2.125	0.01856	0.006 **
**CEC**	1	0.2242	0.22415	1.3421	0.01173	0.146
**Silt**	1	0.138	0.13798	0.8261	0.00722	0.663
**Clay**	1	0.1876	0.18764	1.1234	0.00981	0.308
**Sand**	1	0.1643	0.16429	0.9836	0.00859	0.438
**N**	1	0.262	0.26203	1.5688	0.01371	0.065
**pH**	1	0.275	0.27495	1.6462	0.01438	0.042 *
**EC**	1	0.1707	0.17066	1.0218	0.00893	0.417
**Residuals**	77	12.8605	0.16703		0.67271	
**Total**	96	19.1175			1.00000	

Signif. codes:

* p≤0.05,

** p≤0.01,

*** p≤0.001

OC = organic carbon, avP = available phosphorus, CEC = cation exchange capacity, N = total nitrogen, EC = electrical conductivity.

Among the constraining environmental variables, altitude had the highest bi-plot score value (0.92) in the first ordination axis (CCA1), followed by pH with a bi-plot score of 0.58. Correspondingly, the slope had the highest bi-plot score value (0.76) in the second ordination axis (CCA2), followed by disturbance with a bi-plot score of 0.48. All significant environmental variables were negatively correlated with CCA2, except altitude and OC, which were positively correlated with CCA2. Among the negatively correlated variables, slope had a strong negative correlation with CCA2 (*r = -0*.*76*, *p≤0*.*05)*, followed by disturbance (*r = -0*.*48*, *p≤0*.*05*) (Table 3). The eigenvalue for the first axis was 0.21, and 0.1 for the second axis, showing a declining pattern towards the other consecutive axes. About 96% of the cumulative proportion of variance (total inertia) was explained by the first six axes in the constrained biplot. The first axis (CCA) explained about 40% of the proportion of variance, followed by the second axis (CCA2), which explained about 20% of the total variance. The first two axes (CCA1 and CCA2) together explained about 60% of the total variance (inertia).

**Table 3:** Biplot scores for the constraining variables, eigenvalues and proportion of variances explained by the first six axes.

Variables	CCA1	CCA2	CCA3	CCA4	CCA5	CCA6
**Altitude**	-0.92	0.33	-0.15	-0.05	-0.15	0.08
**Slope**	-0.27	-0.76	-0.30	0.08	-0.46	-0.22
**Disturbance**	0.07	-0.48	0.49	-0.31	0.26	0.60
**OC**	-0.16	0.19	0.04	0.87	-0.13	0.41
**avP**	0.27	-0.13	-0.66	0.45	0.49	0.19
**pH**	0.58	-0.06	-0.38	-0.06	-0.62	0.36
**Eigenvalue**	0.21	0.10	0.07	0.06	0.05	0.04
**Proportion Explained**	0.40	0.19	0.14	0.11	0.09	0.07
**Cumulative Proportion**	0.40	0.59	0.73	0.84	0.93	0.96

#### Relationship between plant community types and environmental variables

Based on the results obtained from the Kruskal-Wallis test, the five plant communities differ significantly from each other with respect to altitude (*X*^*2*^ = 63.99, *p<0*.*001*), slope (*X*^*2*^ = 22.52, *p<0*.*001*), soil pH (*X*^*2*^ = 24.17, *p<0*.*001*) and anthropogenic disturbances (*X*^*2*^ = 24.84, *p<0*.*001*) (Table 4). Dunn’s post-hock pairwise comparison test results revealed that plant community types differ significantly from each other with respect to various environmental factors (S3 Table). For instance, community types 1 and 4, 1 and 5, 2 and 3, 2 and 5, 3 and 4, and 3 and 5 differ significantly with respect to altitude, and community types 1 and 4, 3 and 4, and 4 and 5 differ significantly with disturbance (*p<0*.*05*).

**Table 4:** Kruskal-Wallis test.

	Altitude	Slope	Disturbance	OC	avP	pH
***X*** ^***2***^	63.991	22.522	24.839	4.336	4.487	24.168
**df**	4	4	4	4	4	4
***p-value***	0.000***	0.000***	0.000***	0.362	0.344	0.000***

Signif. codes:

* p≤0.05,

** p≤0.01,

*** p≤0.001

OC = organic carbon, avP = available phosphorus

#### Diversity and evenness of plant community types

We found different Shannon’s diversity index values ranging from 1.23 to 2.60 with a mean value of 2.16, and evenness values ranging from 0.27 to 0.59 with a mean value of 0.49 (Table 5). The descriptive statistical analysis of the topographic and soil variables of each community is given in S4 Table.

**Table 5:** Species richness, evenness and diversity of each community type.

	C1	C2	C3	C4	C5	Mean±Sd
**Species richness**	91	66	58	99	105	83.8±20.7
**Shannon diversity**	1.23	2.34	2.32	2.31	2.6	2.16±0.53
**Shannon evenness**	0.27	0.56	0.57	0.50	0.56	0.49±0.13

C1 = Community type 1, C2 = Community type 2, C3 = Community type 3, C4 = Community type 4, C5 = Community type 5.

## Discussion

We found relatively higher plant species richness (180 vascular plant species) in the study forest compared to other similar studies conducted in Ethiopia, e.g., the vegetation of Chencha highlands in Southern Ethiopia (174 species) [29], Combretum-Terminalia mixed Acacia vegetation in Ilu Gelan District, Central Ethiopia (106 species) [30], and the vegetation of the western escarpment of the rift valley of the Gamo Zone, Southern Ethiopia (126 species) [31]. It is comparable with the findings of [32] for Komoto Forest (180 species) and [33] for dry Afromontane forest patches in Northwestern Ethiopia (176 species). However, the species richness of the study forest is lower than those reported by [34] for the vegetation of the Gambela region in southwestern Ethiopia (220 species) [35], for Key Afer-Shala Luqa and Southwest of Lake Chamo (216 species), and [36] for the natural vegetation in Abaya Hamassa (315 species). These variations in species richness among the forests could be attributed to several factors, such as the altitudinal range in which forests occur, the extent of the sampled area, climate and topographic variability, environmental heterogeneity, regeneration success, competition, and the degree of anthropogenic forest disturbances [37–41].

Poaceae and Fabaceae are the most dominant families in the present study site. These families are the most species-rich families, ranked second and first in the Flora areas of Ethiopia and Eritrea [41] and also world-wide [14]. The dominance of the Poaceae family may be attributed to the ability of the C_4_ grass species to thrive under high moisture stress for most of the year and to withstand disturbance. The dominance of the Fabaceae family may be attributed to the species’ ability to adapt to various ecological conditions, such as resistance of the seeds to predation, recovery of the leaves and branches from defoliation and the rapid germination of seeds when moisture is available and their symbiotic properties [42–44].

### Plant community types

Based on the agglomerative hierarchical cluster analysis and MRRP test results, five significantly different (*p ≤ 0*.*001*) plant community types were identified at the study site. The within-group agreement statistic (A) value among the community types is 0.11. The P-value and A-value are crucial for testing within-group heterogeneity [45]. The A-value falls between 0 and 1, and it defines within-group homogeneity (0 indicates heterogeneity, and 1 indicates the homogeneity of items within clusters). According to [45], an A-value > 0.3 is fairly high in community ecology. The within-group agreement statistics (A) value of the community types in this study, which is far less than 0.3, again indicates the heterogeneity of the vegetation and the distinctness of the clusters. This could be attributed to various constraining environmental factors influencing the variation in species composition among plant communities [46] and anthropogenic disturbances [47].

Based on the CCA analysis results, the plant communities of the study forest were highly influenced by the topographic variables (altitude and slope), edaphic variables (soil organic carbon, soil available phosphorus, and soil pH), and anthropogenic disturbances. The CCA axis 1 is correlated with altitude and pH, while axis 2 is correlated with slope and disturbance. The first axis (CCA) explained about 40% of the proportion of variance, followed by the second axis (CCA2), which explained about 20% of the total variance. Together, CCA1 and CCA2 explained about 60% of the total variance (inertia). This revealed that altitude and pH play a key role in patterns of plant species distribution and community formation in the present study, followed by slope and disturbance, which are correlated to axis 2. This is in agreement with several findings that depict the significant impact of topographic and soil variables on plant species distribution and community formation [3, 19, 48–52].

Besides its overlapping effect among some community types, altitude contributed a lion’s share and is one of the decisive topographic variables in determining the patterns of plant species distribution and plant community formation in the present study. This might be attributed to the fact that variation in altitude causes variations in atmospheric pressure, humidity, temperature, soil type, and microclimatic conditions, which determine the patterns of species by determining the growth and development of plants [1, 8, 9]. Also, altitude as the key topographic or environmental variable in determining patterns of vegetation distribution was reported in similar studies conducted in Ethiopia in different regions of the country by various researchers [16–19, 52, 53].

Slope, the other topographic variable, also had an impact on species distribution and community formation in this study since it is positively correlated with altitude. As pointed out by [3], slope has a strong influence on determining the soil’s chemical and physical properties, and it indirectly shapes the water retention properties of soil depending on the degree of steepness of the environment. [10], also reported the importance of slope in determining plant distribution by causing the accumulation and export of soil nutrients.

Edaphic factors (soil variables) such as avP, OC, and pH also had a crucial impact on the pattern of plant species distribution and community formation in the present study, besides topographic variables. For instance, phosphorus is required in relatively large amounts by plants, and its concentration and availability determine soil fertility and site productivity, which in turn affect plant community formation and distribution [8, 11, 33, 54].

Anthropogenic disturbance (tree cutting and charcoal production) is also an important factor that affects the patterns of species distribution and community formation in addition to the topographic and edaphic variables in this study. This might be attributed to changes in species richness, diversity, distribution patterns, and vegetation structure in an ecosystem as a result of disturbance [3]. [55] also pointed out that disturbance reduces natural regeneration and seedling formation and, hence, affects plant community distribution patterns in tropical forests.

Kruskal-Wallis and Dunn’s pair-wise comparison test indicated that the five plant communities significantly varied from each other with respect to altitude, slope, pH, and disturbance (*p < 0*.*05*) (S3 Table).

We found different diversity index values for each community type, with a mean value of 2.16, which is within the acceptable range of Shannon-Wiener diversity ranging between 1.5 and 3.5 and rarely exceeding 4.5 [22], and generally low evenness values for all community types, which is striking. Community diversity is the outcome of the proportion of the cover abundance of species and the species richness in the community. Both the richness and cover abundance of species in communities can be influenced by the presence and intensity of anthropogenic factors when all other factors are the same. But when multiple factors act on the vegetation cover of an area, the effect of one factor may be strengthened or annulled by the effect of another factor. Thus, the variation in species richness, diversity, and evenness among plant community types could not be attributed to one factor alone, while the combined effect of the various environmental factors (such as altitude, slope), the intensity of anthropogenic disturbances, and other biotic and abiotic factors played their specific roles [46, 47, 56]. The high intensity of disturbance in community types 1 and 3 is manifested by low evenness in the first and low species richness in the second. Community type 3 has a higher diversity (2.32) than community type 4 (2.31), which is in a better state of disturbance. Low diversity can occur at both ends of the disturbance trajectory. High intensity of disturbance, which may result in the removal of high canopy species and the proliferation of weedy or disturbance-favored species. Low disturbance can lead to the dominance of a few tree species and suppress understory species, thus resulting in low diversity. Both community types 1 and 4 have low diversity but different disturbance regimes and species compositions. The Shannon-Wiener diversity index should, therefore, be interpreted with caution and should be related to the species composition and history of the site under consideration.

## Conclusions

The purpose of this study was to determine plant diversity, community types and identify the factors that affect the plant community types in Geramo forest. Five statistically distinct (*p ≤ 0*.*001*) plant community types were identified based on the agglomerative hierarchical cluster analysis and MRRP test results at the study site. Each community type showed different diversity index values and generally low evenness values. Among the thirteen environmental factors examined, only six (altitude, slope, disturbance, organic carbon, available phosphorus, and pH) demonstrated a significant influence on the variation in species composition and community formation. Altitude emerged as the primary environmental variable, followed by disturbance, slope and pH, in determining the differences in species composition and community structure among the community types. Thus, it is better to consider the compounded effect of the various environmental factors to better understand variation in species richness, diversity and evenness among plant community types, as well as plant species distribution in a given area. The study also identified a significant level of anthropogenic disturbance and a strong reliance of the local community on the forest in the research area. Therefore, it is crucial to implement sustainable forest conservation activities that involve the active engagement and participation of the local communities in the surrounding areas with the aim of conserving the forest resources and protecting them from the mounting anthropogenic pressures across different altitudinal ranges.

## Supporting information

S1 Table List of plant species recorded from the study site. (DOCX)

S2 Table Synoptic cover abundance value of species in each community type. (DOCX)

S3 Table Pairwise comparison of clusters using Dunn’s test. (DOCX)

S4 Table Descriptive statistical analysis of the topography and soil variables of each community. (DOCX)
