# Supplementary material for: Determinants of plant community along environmental gradients in Geramo forest, the western escarpment of the rift valley of Ethiopia
Source: PLoS One. 2023 Nov 27;18(11):e0294324. doi: 10.1371/journal.pone.0294324 (PMC10681247; doi:10.1371/journal.pone.0294324)
Supplement: S1 Table — (DOCX) [file pone.0294324.s001.docx]

**S1 Table. List of plant species recorded from the study site**

| **S. No** | **Scientific name** | **Family** | **Habit** |
| --- | --- | --- | --- |
| 1 | *Abrus precatorius* L. | Fabaceae | C |
| 2 | *Acalypha fruticosa* Forssk. | Euphorbiaceae | S |
| 3 | *Acalypha villicaulis* A. Rich. | Euphorbiaceae | H |
| 4 | *Achyranthes aspera* L. | Amaranthaceae | H |
| 5 | *Acokanthera schimperi*(A. DC.) Schweinf. | Apocynaceae | S/T |
| 6 | *Actinopteris semiflabellata* Pic.Serm. | Actinopteridaceae | H |
| 7 | *Adenia gummifera* (Harvey) Harms. | Passifloraceae | C |
| 8 | *Agave sisalana* Perrine ex Engl. | Agavaceae | H |
| 9 | *Allophylus rubifolius* (Hochst. ex A. Rich). Engl. | Sapindaceae | S/T |
| 10 | *Aloe friisii* Sebsebe & M.G. Gilbert | Aloaceae | S |
| 11 | *Aloe megalacantha* Baker | Aloaceae | S |
| 12 | *Aloe otallensis* Bake*r* | Aloaceae | S |
| 13 | *Ampelocissus bombycina* (Bak.) Planch. | Vitaceae | C |
| 14 | *Ampelocissus schimperiana* (Hochst. ex A. Rich.) Planch. | Vitaceae | C |
| 15 | *Anemia schimperiana* C. Presl | Anemiaceae | H |
| 16 | *Aristida adscensionis* L. | Poaceae | H |
| 17 | *Asparagus africanus* Lam. | Asparagaceae | S |
| 18 | *Asparagus falcatus* L. | Asparagaceae | S |
| 19 | *Aspilia mossambicensis* (Oliv.) Wild | Asteraceae | H |
| 20 | *Asplenium* species | Aspleniaceae | H |
| 21 | *Balanites aegyptiaca* (L.) Del. | Balanitaceae | T |
| 22 | *Balanites rotudifolia* (van Tieghm) Blatter | Balanitaceae | S/T |
| 23 | *Baphia abyssinica* Brummitt | Fabaceae | T |
| 24 | *Barleria eranthemoides* R. Br. ex C. B. Clarke | Acanthaceae | S |
| 25 | *Bidens macroptera* (Sch.-Bip. ex Chiov.) Mesfin | Asteraceae | H |
| 26 | *Bidens pilosa* L. | Asteraceae | H |
| 27 | *Boscia angustifolia* A. Rich. | Capparidaceae | T |
| 28 | *Bothriochloa insculpta* (Hochst. ex A. Rich) A. Comus. | Poaceae | H |
| 29 | *Brachiaria deflexa* (Schumach.) Robyns | Poaceae | H |
| 30 | *Bridelia scleroneura* Muell. Arg. | Euphorbiaceae | S |
| 31 | *Cadaba farinosa* Forssk. | Capparidaceae | S |
| 32 | *Calpurnia aurea* (Ait.) Benth. | Fabaceae | S |
| 33 | *Canthium pseudosetiflorum* Bridson | Rubiaceae | S |
| 34 | *Capparis fascicularis* DC. | Capparidaceae | C |
| 35 | *Capparis tomentosa* Lam. | Capparidaceae | C |
| 36 | *Caralluma speciosa* (N.E. Br.) N.E. Br. | Asclepiadaceae | H |
| 37 | *Carissa spinarum* L. | Apocynaceae | Cl |
| 38 | *Chasmanthera dependens* Hochst. | Menispermaceae | C |
| 39 | *Chrysopogon plumulosus* Hochst. | Poaceae | H |
| 40 | *Cissus quadrangularis* L. | Vitaceae | C |
| 41 | *Cissus rotundifolia* (Forssk.) Vahl | Vitaceae | C |
| 42 | *Combretum collinum* Fresen. | Combretaceae | T |
| 43 | *Combretum molle* R. Br. ex G. Don. | Combretaceae | T |
| 44 | *Combretum species* | Combretaceae | T |
| 45 | *Commelina diffusa* Burm. f | Commelinaceae | H |
| 46 | *Commelina erecta* L. | Commelinaceae | H |
| 47 | *Commiphora africana* (A. Rich.) Engl. | Burseraceae | T |
| 48 | *Commiphora habessinica* (Berg) Engl. | Burseraceae | T |
| 49 | *Commiphora schimperi* (Berg) Engl. | Burseraceae | T |
| 50 | *Conyza bonariensis* (L.) Cronq. | Asteraceae | H |
| 51 | *Cordia monoica* Roxb. | Boraginaceae | S |
| 52 | *Crossandra massaica* Mildbr. | Acanthaceae | S |
| 53 | *Crotalaria incana* L. | Fabaceae | H |
| 54 | *Crotalaria laburnifolia* L. | Fabaceae | H |
| 55 | Croton zambesicus Muell. Arg. | Euphorbiaceae | T |
| 56 | *Cyathula orthacantha* (Aschers.) Schinz | Amaranthaceae | H |
| 57 | *Cynodon dactylon* (L.) Pers. | Poaceae | H |
| 58 | *Cyperus dubius* Rottb. | Cyperaceae | H |
| 59 | *Cyperus microstylis* (C.B. Clarke) Mattf. & Kuk | Cyperaceae | H |
| 60 | *Cyperus rotundus* L. | Cyperaceae | H |
| 61 | *Cyphostemma adenocaule* (Steud. ex A. Rich.) Desc. ex Wild & Drummond | Vitaceae | C |
| 62 | *Cyphostemma species* Gilbert & Thulin | Vitaceae | C |
| 63 | *Dactyloctenium aegyptium* (L.) Willd. | Poaceae | H |
| 64 | *Dichrostachys cinerea* (L.) Wight & Arn. | Fabaceae | S |
| 65 | *Digitaria abyssinica* (Hochst. Ex A. Rich.) Stapf | Poaceae | H |
| 66 | *Digitaria ternata* (A. Rich.) Stapf | Poaceae | H |
| 67 | *Diospyros abyssinica* (Hiern) F. White | Ebenaceae | T |
| 68 | *Dodonaea angustifolia* L. f. | Sapindaceae | S |
| 69 | *Echinochloa colona* (L.) Link | Poaceae | H |
| 70 | *Ehretia cymosa* Thonn. | Boraginaceae | T |
| 71 | *Elaeodendron buchananii* (Loes.) Loes. | Celastraceae | T |
| 72 | *Erythrina abyssinica* Lam. ex DC. | Fabaceae | T |
| 73 | *Euclea divinorum* Hiern. | Ebenaceae | T |
| 74 | *Eulalia species* | Poaceae | H |
| 75 | *Euphorbia ampliphylla* Pax | Euphorbiaceae | T |
| 76 | *Euphorbia nigrispina* N. E. Br. | Euphorbiaceae | S |
| 77 | *Euphorbia tirucalli* L. | Euphorbiaceae | T |
| 78 | *Ficus sycomorus* L. | Moraceae | T |
| 79 | *Ficus vasta* Forsk. | Moraceae | T |
| 80 | *Flacourtia indica* (Burm. f.) Merr. | Flacourtiaceae | T |
| 81 | *Flueggea virosa* (Willd.) Voigt. | Euphorbiaceae | S |
| 82 | *Geigeria alata* (DC.) Benth. & Hook. f. ex Oliv. & Hiern | Asteraceae | H |
| 83 | *Gomphocarpus fruticosus* (L.) Ait. f. | Asclepiadaceae | H |
| 84 | *Grewia bicolor* Juss. | Tiliaceae | S/T |
| 85 | *Grewia erythraea* Schweinfurth | Tiliaceae | S |
| 86 | *Grewia flavescens* Juss. | Tiliaceae | C |
| 87 | *Grewia velutina* (Forssk.) Vahl | Tiliaceae | S/T |
| 88 | *Grewia villosa* Willd. | Tiliaceae | S |
| 89 | *Guizotia species* | Asteraceae | H |
| 90 | *Harrisonia abyssinica* Oliv. | Simaroubaceae | S |
| 91 | *Heteropogon contortus* (L.) Roem. & Schult. | Poaceae | H |
| 92 | *Heteropogon melanocarpus* (Ell.) Benth. | Poaceae | H |
| 93 | *Hippocratea pallens* Planch. ex Oliver | Celastraceae | S |
| 94 | *Hyparrhenia filipendula* (Hochst.) Stapf | Poaceae | H |
| 95 | *Hyparrhenia hirta* (L.) Stapf | Poaceae | H |
| 96 | *Indigofera schimperi* Jaub. & Spach | Fabaceae | H |
| 97 | *Jasminum grandiflorum* L. | Oleaceae | C |
| 98 | *Justicia flava* (Vahl) Vahl | Acanthaceae | H |
| 99 | *Justica ladanoides* Lam. | Acanthaceae | H |
| 100 | *Kalanchoe glaucescens* Britten | Crassulaceae | H |
| 101 | *Kalanchoe lanceolata* (Forssk.) Pers. | Crassulaceae | H |
| 102 | *Kleinia squarrosa* Cufod. | Asteraceae | C |
| 103 | *Lannea barteri* (Oliv.) Engl. | Anacardiaceae | T |
| 104 | *Lannea schimperi* (A. Rich.) Engl. | Anacardiaceae | T |
| 105 | *Lantana camara* L. | Verbenaceae | S |
| 106 | *Leonotis ocymifolia* (Burm. f.) Iwarsson | Lamiaceae | H |
| 107 | *Lepidotrichilia volkensii* (Gurke) Leroy | Meliaceae | T |
| 108 | *Leptochloa uniflora* Hochst. ex A. Rich. | Poaceae | H |
| 109 | *Leucas abyssinica* (Benth.) Briq. | Lamiaceae | S |
| 110 | *Leucas martinicensis* (Jacq.) R. Br. | Lamiaceae | H |
| 111 | *Maerua angolensis* DC. | Capparidaceae | S |
| 112 | *Maerua crassifolia* Forssk. | Capparidaceae | T |
| 113 | *Maytenus senegalensis* (Lam.) Exell | Celastraceae | S |
| 114 | *Maytenus undata* (Thunb.) Blakelock | Celastraceae | T |
| 115 | *Melhania* species | Sterculiaceae | H |
| 116 | *Melinis repens* (Willd.) Zizka | Poaceae | H |
| 117 | *Melinis* *tenuissima* Stapf | Poaceae | H |
| 118 | *Monanthotaxis parvifolia* (Oliv.) Verdc. | Annonaceae | S |
| 119 | *Mystroxylon aethiopicum* (Thunb.) Loes. | Celastraceae | T |
| 120 | *Nuxia oppositifolia* (Hochst.) Benth. | Loganiaceae | S |
| 121 | *Ocimum canum* Sims | Lamiaceae | H |
| 122 | *Oldenlandia* species | Rubiaceae | H |
| 123 | *Olea europaea* L. *subsp. cuspidata (*Wall. ex G. Don) Cif. | Oleaceae | T |
| 124 | *Opuntia ficus-indica* (L.) Miller | Cactaceae | S |
| 125 | *Osyris quadripartita* Decn. | Santalaceae | T |
| 126 | *Ozoroa insignis* Del. | Anacardiaceae | T |
| 127 | *Panicum monticola* Hook.f. | Poaceae | H |
| 128 | *Pappea capensis* Eckl. & Zeyh. | Sapindaceae | T |
| 129 | *Pavetta gardenifolia* A. Rich. | Rubiaceae | S |
| 130 | *Pavonia sp.* Friis et al. 2798 | Malvaceae | S |
| 131 | *Pelargonium multibracteatum* Hochst. ex A. Rich. | Geraniaceae | H |
| 132 | *Pellaea calomelanos* (Sw.) Link | Pteridaceae | H |
| 133 | *Pennisetum glabrum* Steud. | Poaceae | H |
| 134 | *Pennisetum nubicum* (Hochst.) K. Schum. Ex Engl. | Poaceae | H |
| 135 | *Pennisetum pedicellatum* Trin. | Poaceae | H |
| 136 | *Pergularia daemia* (Forssk.) Chiov. | Asclepiadaceae | C |
| 137 | *Perotis patens* Gand. | Poaceae | H |
| 138 | *Phragmites karka* (Retz.) Steud. | Poaceae | H |
| 139 | *Plectranthus comosus* Sims | Lamiaceae | H |
| 140 | *Rhoicissus revoilii* Planch. | Vitaceae | C |
| 141 | *Rhoicissus tridentata* (L. f.) Wild & Drummond | Vitaceae | C |
| 142 | *Rhus natalensis* Krauss | Anacardiaceae | S |
| 143 | *Rhynchosia stipulosa* A. Rich. | Fabaceae | C |
| 144 | *Ricinus communis* L. | Euphorbiaceae | S |
| 145 | *Sansevieria ehrenbergii* Schweinf. ex Baker | Dracaenaceae | S |
| 146 | *Sansevieria forskaoliana* (Schult. f.) Hepper & Wood | Dracaenaceae | S |
| 147 | *Sansevieria nilotica* Baker | Dracaenaceae | S |
| 148 | *Sarcostemma viminale* (L.) R. Br. | Asclepiadaceae | C |
| 149 | *Schlechterella abyssinica* (Chiov.) Venter & R. L. Verh. | Asclepiadaceae | C |
| 150 | *Sclerocarya birrea* (A. Rich.) Hochst. | Anacardiaceae | T |
| 151 | *Senegalia brevispica* (Harms) Seigler & Ebinger | Fabaceae | S |
| 152 | *Senegalia senegal* (L.) Britton | Fabaceae | T |
| 153 | *Senna didymobotrya* (Fresen.) Irwin & Barneby | Fabaceae | S |
| 154 | *Setaria megaphylla* (Steud.) Th. Dur. & Schinz | Poaceae | H |
| 155 | *Setaria pumila* (Poir.) Roem. & Schult. | Poaceae | H |
| 156 | *Setaria sphacelata* (Schumach.) Moss | Poaceae | H |
| 157 | *Solanum incanum* L. | Solanaceae | S |
| 158 | *Sorghum arundinaceum* (Desv.) Stapf | Poaceae | H |
| 159 | *Sporobolus festivus* Hochst. ex A. Rich. | Poaceae | H |
| 160 | *Sporobolus ioclados* (Trin.) Nees | Poaceae | H |
| 161 | *Steganotaenia araliacea* Hochst. ex A. Rich. | Apiaceae | T |
| 162 | *Stereospermum kunthianum* Cham. | Bignoniaceae | T |
| 163 | *Stylosanthes fruticosa* (Retz.) Alston | Fabaceae | H |
| 164 | *Tamarindus indica* L. | Fabaceae | T |
| 165 | *Teclea nobilis* Del. | Rutaceae | S/T |
| 166 | *Terminalia brownii* Fresen. | Combretaceae | T |
| 167 | *Terminalia schimperiana* Hochst. | Combretaceae | T |
| 168 | *Tricalysia niamniamensis* Hiern | Rubiaceae | S |
| 169 | *Vachellia dolichocephala* (Harms) Kyal. & Boatwr. | Fabaceae | T |
| 170 | *Vachellia nilotica* (L.) P.J.H. Hurter & Mabb. | Fabaceae | T |
| 171 | *Vachellia seyal* (Delile) P.J.H. Hurter | Fabaceae | T |
| 172 | *Vachellia tortilis* (Forssk.) Galasso & Banfi | Fabaceae | T |
| 173 | *Vernonia cinerascens* Sch. Bip. in Schweinf. & Asch. | Asteraceae | S |
| 174 | *Ximenia americana* L. | Olacaceae | S |
| 175 | *Ximenia caffra* Sond. | Olacaceae | S |
| 176 | *Zaleya pentandra (L.) Jeffrey* | Aizoaceae | H |
| 177 | *Zanthoxylum chalybeum* Engl. | Rutaceae | T |
| 178 | *Ziziphus mucronata* Willd. | Rhaminaceae | S/T |
| 179 | *Ziziphus spina-christi* (L.) Desf. | Rhaminaceae | T |
| 180 | *Zornia setosa* Bak.f. | Fabaceae |  |

*T = Tree, S = Shrub, C = Climber, H = Herb.*
