# Supplementary material for: Determinants of plant community along environmental gradients in Geramo forest, the western escarpment of the rift valley of Ethiopia
Source: PLoS One. 2023 Nov 27;18(11):e0294324. doi: 10.1371/journal.pone.0294324 (PMC10681247; doi:10.1371/journal.pone.0294324)
Supplement: S2 Table — (DOCX) [file pone.0294324.s002.docx]

**S2 table. Synoptic cover abundance value of species in each community type**

| **Species** | **C 1** | **C 2** | **C 3** | **C 4** | **C 5** |
| --- | --- | --- | --- | --- | --- |
| *Dichrostachys cinerea* | **5.05** | 5.01 | 3.4 | 3 | 4.26 |
| *Balanites aegyptiaca* | **3.79** | 2.57 | 3.62 | 1.67 | 3.21 |
| *Commiphora africana* | **3.53** | 4.71 | 3.49 | 2.27 | 1.05 |
| *Grewia bicolor* | 3.07 | 2.79 | 1.2 | 3 | 2.26 |
| *Euclea divinorum* | 3 | 0.79 | 0 | 1.6 | 2.76 |
| *Tamarindus indica* | 3.5 | 2.95 | 2 | 2.67 | 2.32 |
| *Nuxia oppositifolia* | 0.11 | 0 | 0 | 0 | 0 |
| *Vernonia cinerascens* | 0.37 | 0 | 0 | 0 | 0 |
| *Bothriochloa insculpta* | 0.11 | 0 | 0 | 0 | 0 |
| *Commelina erecta* | 0.21 | 0 | 0 | 0 | 0 |
| *Setaria megaphylla* | 0.16 | 0 | 0 | 0 | 0 |
| *Echinochloa colona* | 0.16 | 0 | 0 | 0 | 0 |
| *Ziziphus spina-christi* | 0.16 | 0 | 0 | 0 | 0 |
| *Ficus vasta* | 0.26 | 0 | 0 | 0 | 0 |
| *Ximenia cafra* | 0.42 | 0 | 0 | 0 | 0 |
| *Sansevieria nilotica* | 0.42 | 0 | 0 | 0 | 0 |
| *Euphorbia ampliphylla* | 0.21 | 0 | 0 | 0 | 0 |
| *Euphorbia nigrispina* | 0.05 | 0 | 0 | 0 | 0 |
| *Pergularia daemia* | 0.05 | 0 | 0 | 0 | 0 |
| *Schlechterella abyssinica* | 0.21 | 0 | 0 | 0 | 0 |
| *Cyphostemma adenocaule* | 0.21 | 0 | 0 | 0 | 0 |
| *Leucas abyssinica* | 0.29 | 0 | 0 | 0 | 0 |
| *Kleinia squarrosa* | 0.63 | 0 | 0.3 | 0.07 | 0.05 |
| *Asparagus falcatus* | 0.16 | 0 | 0 | 0.07 | 0 |
| *Cyperus dubius* | 0.37 | 0 | 0 | 0.27 | 0 |
| *Cissus rotundifolia* | 0.4 | 0 | 0.37 | 0.07 | 0 |
| *Cyathula orthacantha* | 0.57 | 0 | 0 | 0.27 | 0.32 |
| *Cissus quadrangularis* | 0.63 | 0 | 0.4 | 0.33 | 0 |
| *Terminalia brownii* | 3.26 | **7.64** | 1.5 | 3.37 | 4.26 |
| *Hyparrhenia filipendula* | 2.53 | **7.14** | 4.4 | 2.73 | 4.30 |
| *Rhus natalensis* | 3.01 | **6.36** | 4.3 | 3.93 | 4.29 |
| *Sclerocarya birrea* | 1.58 | 4.64 | 2.1 | 1.67 | 0.68 |
| *Ximenia americana* | 2.47 | 3.14 | 2.6 | 1.13 | 1.76 |
| *Commiphora schimperi* | 0 | 3.14 | 0 | 0.47 | 0.26 |
| *Ocimum canum* | 0 | 0.74 | 0.7 | 0.33 | 0 |
| *Gomphocarpus fruticosus* | 0 | 0.37 | 0 | 0 | 0.11 |
| *Grewia villosa* | 0.5 | 2.11 | 1.4 | 0.87 | 0 |
| *Lantana camara* | 0.05 | 0.1 | 0 | 0 | 0 |
| *Melinis repens* | 0.16 | 0.6 | 0 | 0 | 0 |
| *Cyperus microstylis* | 0.21 | 0.8 | 0.4 | 0 | 0 |
| *Sarcostemma viminale* | 0.36 | 0.79 | 0.3 | 0.67 | 0.13 |
| *Balanites rotudifolia* | 1.11 | 2.6 | 1.01 | 1 | 0.05 |
| *Euphorbia tirucalli* | 0.16 | 0.2 | 0 | 0.4 | 0 |
| *Barleria eranthemoides* | 2.39 | 2.79 | 0.5 | 1.67 | 0.71 |
| *Cynodon dactylon* | 0 | 2.8 | 1.8 | 0 | 0 |
| *Pavetta gardenifolia* | 0 | 1.05 | 0.4 | 0.73 | 0.16 |
| *Sansevieria forskaoliana* | 0 | 0.1 | 0 | 0 | 0 |
| *Solanum incanum* | 0.05 | 0.13 | 0 | 0 | 0 |
| *Sansevieria ehrenbergii* | 0.11 | 0.27 | 0 | 0 | 0 |
| *Indigofera schimperi* | 1 | 1.5 | 0 | 1.2 | 0.47 |
| *Canthium pseudosetiflorum* | 2.71 | 2.89 | 0.6 | 2.07 | 1.34 |
| *Acalypha fruticosa* | 1.74 | 2.4 | 1.6 | 0.43 | 0.53 |
| *Aspilia mossambicensis* | 0 | 1.1 | 0 | 0 | 0 |
| *Flueggea virosa* | 0.71 | 0.95 | 1.2 | 0.27 | 0.42 |
| *Maerua crassifolia* | 0 | 0.11 | 0 | 0 | 0 |
| *Grewia erythraea* | 0 | 0.4 | 0 | 0 | 0 |
| *Vachellia nilotica* | 3.11 | 5.64 | **6** | 1.87 | 1.76 |
| *Harrisonia abyssinica* | 2.05 | 4.86 | **4.9** | 1.27 | 4.19 |
| *Ziziphus mucronata* | 2.68 | 1.57 | **4.5** | 1.13 | 2.21 |
| *Senegalia senegal* | 2.74 | 0.5 | 4.4 | 1.27 | 0.24 |
| *Brachiaria deflexa* | 0.58 | 0 | 3.3 | 0 | 0.11 |
| *Commelina diffusa* | 0.16 | 0 | 3.1 | 0.67 | 0 |
| *Achyranthes aspera* | 0 | 0 | 0.13 | 0 | 0 |
| *Hyparrhenia filipendula* | 0.21 | 0 | 0.29 | 0 | 0.13 |
| *Erythrina abyssinica* | 0.21 | 0 | 0.67 | 0 | 0.11 |
| *Commiphora habessinica* | 0.26 | 0 | 2.71 | 0.87 | 0.45 |
| *Lepidotrichilia volkensii* | 0 | 0.14 | 0.42 | 0.33 | 0.11 |
| *Justicia flava* | 0 | 0 | 0.4 | 0 | 0 |
| *Grewia flavescens* | 1.5 | 0.79 | 1.63 | 1.07 | 0.87 |
| *Anemia schimperiana* | 0 | 0 | 0.43 | 0.4 | 0 |
| *Stylosanthes fruticosa* | 0.95 | 1.6 | 2.01 | 0.93 | 0.95 |
| *Actinopteris semiflabellata* | 0 | 1 | 1.6 | 0 | 0.08 |
| *Cyperus rotundus* | 0 | 0 | 1.5 | 0 | 0.11 |
| *Capparis fascicularis* | 0 | 0 | 0.42 | 0.27 | 0.24 |
| *Vachellia seyal* | 0.68 | 0 | 1.86 | 0.53 | 1.45 |
| *Cordia monoica* | 0 | 0 | 0.8 | 0.7 | 0 |
| *Abrus precatorius* | 0 | 0 | 0.2 | 0 | 0 |
| *Asparagus africanus* | 0 | 0 | 0.11 | 0 | 0.11 |
| *Boscia angustifolia* | 1.32 | 0.64 | 1.8 | 0.8 | 1.5 |
| *Vachellia tortilis* | 0 | 0.29 | 2.6 | 0.53 | 0.32 |
| *Aloe otallensis* | 0 | 0 | 0.07 | 0 | 0 |
| *Acokanthera schimperi* | 0.63 | 0 | 1.4 | 0 | 0.82 |
| *Diospyros abyssinica* | 0.11 | 0.36 | 1.03 | 1 | 0 |
| *Tricalysia niamniamensis* | 1.10 | 0 | 1.12 | 0.67 | 0 |
| *Senegalia brevispica* | 0.53 | 0.57 | 1 | **4.8** | 0.95 |
| *Croton zambesicus* | 0.53 | 0 | 0.5 | **3.8** | 0.18 |
| *Teclea nobilis* | 1.79 | 0.86 | 0 | **3.47** | 2.47 |
| *Pappea capensis* | 2.05 | 1.5 | 0.4 | 3.42 | 3.0 |
| *Panicum monticola* | 0 | 0 | 0.4 | 2.73 | 0.55 |
| *Combretum molle* | 2.37 | 0 | 0 | 2.53 | 0.53 |
| *Crotalaria incana* | 0 | 0 | 0 | 0.27 | 0 |
| *Pavonia species* | 0 | 0 | 0 | 0.27 | 0 |
| *Allophylus rubifolius* | 0.63 | 0.43 | 0.6 | 2 | 1 |
| *Ampelocissus bombycina* | 0 | 0.36 | 0 | 1.2 | 0.13 |
| *Sporobolus festivus* | 0 | 0.67 | 0 | 0.93 | 0.39 |
| *Leptochloa uniflora* | 0.53 | 0.29 | 0 | 1.07 | 0.63 |
| *Phragmites karka* | 0.74 | 0.57 | 0.3 | 3.45 | 1.37 |
| *Bidens pilosa* | 0 | 0 | 0 | 0.36 | 0.26 |
| *Ampelocissus schimperiana* | 0 | 0.07 | 0 | 0.53 | 0.05 |
| *Rhynchosia stipulosa* | 0.26 | 1.71 | 1 | 2.13 | 1.63 |
| *Olea europaea L. subsp. cuspidata* | 0.37 | 0.29 | 0 | 0.68 | 0.33 |
| *Carissa spinarum* | 0.32 | 0 | 0.2 | 0.34 | 0.33 |
| *Eulalia species* | 0 | 0 | 0 | 0.33 | 0.11 |
| *Leucas martinicensis* | 0 | 0.33 | 0 | 0.71 | 0.34 |
| *Baphia abyssinica* | 0 | 0 | 0 | 0.6 | 0 |
| *Steganotaenia araliacea* | 0 | 0 | 0 | 1.33 | 0.26 |
| *Asplenium species* | 0 | 0 | 0 | 0.67 | 0.11 |
| *Jasminum grandiflorum* | 0.11 | 0.21 | 0 | 0.42 | 0 |
| *Kalanchoe glaucescens* | 0 | 0 | 0 | 0.4 | 0.05 |
| *Rhoicissus tridentata* | 0.11 | 0 | 0 | 0.87 | 0.42 |
| *Plectranthus comosus* | 0 | 0.33 | 0 | 1.21 | 0.62 |
| *Setaria pumila* | 0 | 0.93 | 0 | 1.71 | 0.92 |
| *Combretum collinum* | 0.21 | 0.64 | 0 | 1.53 | **7.37** |
| *Heteropogon contortus* | 2.42 | 5.05 | 1.5 | 3 | **5.18** |
| *Grewia velutina* | 1.26 | 3.79 | 0.7 | 1.47 | **4.32** |
| *Dodonaea angustifolia* | 0.47 | 0.57 | 0.2 | 0.2 | 3.39 |
| *Lannea schimperi* | 0.16 | 1.5 | 0 | 2.4 | 3.16 |
| *Sporobolus ioclados* | 0 | 1.29 | 0 | 1 | 2.34 |
| *Cyphostemma species* | 0.11 | 0 | 0 | 0 | 0.26 |
| *Zanthoxylum chalybeum* | 0.16 | 0.29 | 0.3 | 0.8 | 1.08 |
| *Maytenus senegalensis* | 0 | 0.43 | 0.6 | 0 | 0.87 |
| *Chrysopogon plumulosus* | 0.16 | 0 | 0 | 0.24 | 0.4 |
| *Stereospermum kunthianum* | 0.16 | 0 | 0 | 0 | 0.58 |
| *Ozoroa insignis* | 0 | 0.64 | 0 | 0 | 1.13 |
| *Combretum species* | 0.21 | 1.5 | 0.4 | 0.33 | 3.45 |
| *Mystroxylon aethiopicum* | 0 | 0.21 | 0 | 1.2 | 2.08 |
| *Bridelia scleroneura* | 0 | 0.29 | 0 | 0.47 | 1.11 |
| *Osyris quadripartita* | 0 | 0.14 | 0 | 0.33 | 1.87 |
| *Melinis tenuissima* | 0 | 1.07 | 0 | 0.27 | 1.24 |
| *Sorghum arundinaceum* | 0 | 0 | 0 | 0.4 | 0.66 |
| *Heteropogon melanocarpus* | 0 | 0 | 0 | 0.4 | 0.47 |
| *Pennisetum glabrum* | 0 | 0 | 0 | 0.53 | 0.92 |
| *Monanthotaxis parvifolia* | 0 | 0 | 0 | 0 | 0.45 |
| *Flacourtia indica* | 0 | 0 | 0 | 0.33 | 1.39 |
| *Elaeodendron buchananii* | 0 | 0 | 0 | 0 | 0.11 |
| *Pellaea calomelanos* | 0 | 0 | 0 | 0 | 0.08 |
| *Terminalia schimperiana* | 0 | 0 | 0 | 0 | 0.18 |
| *Justica ladanoides* | 0 | 0 | 0 | 0 | 0.11 |
| *Maytenus undata* | 0 | 0 | 0 | 0 | 0.21 |
| *Calpurnia aurea* | 0 | 0 | 0 | 0 | 0.08 |

*C1 = Community type 1, C2 =* *Community type 2, C3 = Community type 3, C4 = Community type 4, C5 = Community type 5*
