# Supplementary material for: Determinants of plant community along environmental gradients in Geramo forest, the western escarpment of the rift valley of Ethiopia
Source: PLoS One. 2023 Nov 27;18(11):e0294324. doi: 10.1371/journal.pone.0294324 (PMC10681247; doi:10.1371/journal.pone.0294324)
Supplement: S3 Table — (DOCX) [file pone.0294324.s003.docx]

**S3 Table.** **Pairwise comparison of clusters using Dunn’s test**

| **Variables** | **Pair-wise clusters** | **Test Statistic** | **Std.Error** | **Std.Test Statistic** | **Sig.** | **Adj.Sig.** |
| --- | --- | --- | --- | --- | --- | --- |
| **Altitude** | 1&4 | -28.65 | 9.62 | -2.98 | 0.003 | 0.029 |
|  | 1&5 | -48.14 | 7.83 | -6.15 | 0.000 | 0.000 |
|  | 2&3 | 38.66 | 11.53 | 3.35 | 0.001 | 0.008 |
|  | 2&5 | -25.85 | 8.71 | -2.97 | 0.003 | 0.030 |
|  | 3&4 | -45.02 | 11.37 | -3.96 | 0.000 | 0.001 |
|  | 3&5 | -64.52 | 9.90 | -6.52 | 0.000 | 0.000 |
| **Disturbance** | 1&4 | 28.42 | 8.39 | 3.39 | 0.001 | 0.007 |
|  | 3&4 | 46.50 | 9.91 | 4.69 | 0.000 | 0.000 |
|  | 4&5 | -28.15 | 7.40 | -3.80 | 0.000 | 0.001 |
| **pH** | 1&5 | 25.53 | 7.83 | 3.26 | 0.001 | 0.011 |
|  | 2&3 | -33.11 | 11.53 | -2.87 | 0.004 | 0.041 |
|  | 3&5 | 43.41 | 9.89 | 4.38 | 0.000 | 0.000 |
| **Slope** | 1&2 | -33.56 | 9.75 | -3.44 | 0.001 | 0.006 |
|  | 2&3 | 46.79 | 11.46 | 4.08 | 0.000 | 0.000 |
|  | 3&4 | -35.62 | 11.29 | -3.15 | 0.002 | 0.016 |
| *P<0.05* | | | | | |  |
