# Supplementary material for: Determinants of plant community along environmental gradients in Geramo forest, the western escarpment of the rift valley of Ethiopia
Source: PLoS One. 2023 Nov 27;18(11):e0294324. doi: 10.1371/journal.pone.0294324 (PMC10681247; doi:10.1371/journal.pone.0294324)
Supplement: S4 Table — (DOCX) [file pone.0294324.s004.docx]

**S4 Table.** **Descriptive statistical analysis of the topography and soil variables of each community**

| **Community** | **Plots** | **Statistics** | **Topographic variables** | | | | | | | | | | | **Diversity** | | |
| --- | --- | --- | --- | --- | --- | --- | --- | --- | --- | --- | --- | --- | --- | --- | --- | --- |
|  |  |  | **Alt** | **Slope** | **SOC** | **avP** | **CEC** | **Sand** | **Clay** | **Silt** | **TN** | **pH** | **EC** | **S** | **H** | **E** |
| 1 | 19 | Min | 1214 | 1 | 1.03 | 0.68 | 11.16 | 19.00 | 20.00 | 15.00 | 0.12 | 6.36 | 0.11 | 91 | 1.23 | 0.27 |
|  |  | Max | 1262 | 50 | 3.01 | 6.04 | 40.40 | 51.00 | 50.00 | 49.00 | 0.27 | 7.50 | 1.00 |  |  |  |
|  |  | Mean | 1235.95 | 15.2 | 1.84 | 2.87 | 30.35 | 35.84 | 35.37 | 28.8 | 0.19 | 7 | 0.45 |  |  |  |
|  |  | Std.Dev | 15.7 | 11.7 | 0.5 | 1.68 | 9.27 | 10.31 | 8.30 | 9.59 | 0.04 | 0.3 | 0.23 |  |  |  |
| 2 | 14 | Min | 1222 | 10 | 1.14 | 1.01 | 19.1 | 33 | 16 | 21 | 0.11 | 6.41 | 0.12 | 66 | 2.34 | 0.56 |
|  |  | Max | 1350 | 70 | 3.46 | 8.73 | 44.64 | 55 | 40 | 51 | 0.31 | 7.47 | 0.98 |  |  |  |
|  |  | Mean | 1281.64 | 46.79 | 1.86 | 2.69 | 31.69 | 44.23 | 24.31 | 31.5 | 0.21 | 6.79 | 0.36 |  |  |  |
|  |  | Std.Dev | 43.93 | 22.24 | 0.65 | 2.32 | 8.02 | 6.51 | 7.06 | 7.96 | 0.06 | 0.29 | 0.23 |  |  |  |
| 3 | 10 | Min | 1199 | 5 | 1.4 | 1.62 | 12.86 | 19 | 22 | 15 | 0.14 | 6.90 | 0.31 | 58 | 2.32 | 0.57 |
|  |  | Max | 1223 | 20 | 2.75 | 4.88 | 34.9 | 55 | 56 | 49 | 0.34 | 7.44 | 1.04 |  |  |  |
|  |  | Mean | 1211.9 | 7.8 | 1.89 | 2.55 | 26.51 | 32.8 | 36.2 | 31 | 0.23 | 7.18 | 0.58 |  |  |  |
|  |  | Std.Dev | 7.79 | 4.78 | 0.42 | 1.07 | 6.28 | 11.52 | 11.29 | 11.8 | 0.07 | 0.19 | 0.27 |  |  |  |
| 4 | 15 | Min | 1235 | 0 | 1.12 | 1.3 | 18.12 | 29 | 18 | 19 | 0.06 | 6.33 | 0.16 | 99 | 2.31 | 0.5 |
|  |  | Max | 1407 | 80 | 4.05 | 4.92 | 41.2 | 55 | 38 | 45 | 0.49 | 7.47 | 0.92 |  |  |  |
|  |  | Mean | 1297.87 | 41.33 | 1.97 | 2.33 | 29.2 | 42.2 | 27.87 | 29.9 | 0.23 | 6.81 | 0.45 |  |  |  |
|  |  | Std.Dev | 51.37 | 27.02 | 0.7 | 0.93 | 7.25 | 8.48 | 5.37 | 7.59 | 0.1 | 0.36 | 0.27 |  |  |  |
| 5 | 38 | Min | 1245 | 0 | 1.12 | 1.19 | 11.44 | 23 | 4 | 7 | 0.11 | 6.20 | 0.08 | 105 | 2.6 | 0.56 |
|  |  | Max | 1439 | 75 | 4.07 | 4.16 | 43.22 | 81 | 40 | 45 | 0.48 | 7.45 | 1.00 |  |  |  |
|  |  | Mean | 1356.21 | 29.24 | 2.01 | 2.02 | 26.48 | 46.35 | 26.81 | 26.8 | 0.22 | 6.66 | 0.29 |  |  |  |
|  |  | Std.Dev | 58.73 | 23.62 | 0.62 | 0.68 | 6.94 | 11.15 | 7.06 | 9.07 | 0.08 | 0.31 | 0.21 |  |  |  |

*Alt = altitude, SOC = soil organic carbon, avP = available phosphorus, CEC = cation exchange capacity, TN = total nitrogen, EC = electrical conductivity, S = species richness, H’ = Shannon’s diversity, E = Shannon’s evenness.*
